# Mortality, hospital days and treatment costs of current and reduced sugar consumption in Israel

**DOI:** 10.1186/s13584-016-0129-9

**Published:** 2017-01-10

**Authors:** Gary M. Ginsberg

**Affiliations:** Israel Ministry of Health, Public Health Services, Yirmiahu Street 39, Jerusalem, 9446724 Israel

**Keywords:** Attributable mortality, Hospitalisations, Sugar, Obesity, Overweight

## Abstract

**Background:**

Consumption of sugar causes tooth decay, overweight and obesity related morbidities. This paper in response to the Minister of Health’s request, provides estimates of the mortality, morbidity and health care costs attributable to sugar consumption in Israel along with the effects of reducing sugar consumption.

**Methods:**

Gender specific relative risks of many diseases from overweight (25 < =BMI < 30) and obesity (BMI > =30) were applied to the national gender specific prevalence rates of overweight and obesity in order to calculate the population attributable fraction (PAF) from overweight and obesity. National expenditure on these related diseases was calculated by applying disease-specific data from a recent Canadian study to estimates of disease specific general hospital expenditures in Israel. Disease specific costs attributable to overweight and obesity were estimated from the product of these expenditures and PAF. In addition national costs of treating caries in persons under 18 years of age from sugar were calculated. Similar calculations were made to estimate the burden from sugar in terms of mortality and hospital utilisation. A recent UK modelling study was used to estimate the effect of a national program to reduce calorific consumption of sugar from 12.45 to 10% in 5 years.

**Results:**

Conditions associated with overweight or obesity accounted annually for 6402 deaths (95% CI 3296–8760) and 268,009 hospital days. Dental costs attributable to sugar consumption were 264 million NIS. In total, obesity, overweight and sugar consumption accounted for 2449 million in direct treatment costs (0.21% of GDP), rising to 4027 million (0.35% of GDP) when indirect costs were included. A national program of reducing energy from sugar consumption from 12.45 to 10% over 5 years is considered have a very feasible short-term goal. Even if the program does not impose taxes on sugar consumption, this would save 778 million NIS as well as 1184 lives.

**Conclusion:**

Sugar consumption causes a huge monetary and mortality burden. Estimates of potential decreases in this burden justify the current prioritisation given by the health minister of creating and implementing a national program to reduce sugar consumption, which is likely to be cost-saving (ie: averted treatment costs will exceed intervention costs).

## Background

The increasing prevalence of obesity, which has more than doubled since 1980 [1] is an important public health problem that contributes to excess morbidity and, to a lesser degree, increased mortality [2]. In 2000, the World Health Organization (WHO) attributed 7–25% of total health care costs worldwide [2] to obesity. In 2014, worldwide, there were over 1.9 billion overweight (25 = <Body Mass Index [BMI] < 30) in addition to more than 600 million obese adults (BMI ≥ 30). Elevated BMI is associated with a higher risk of many non-communicable diseases [1].

Overweight and obesity, as well as their related non-communicable diseases, are largely preventable. It is acknowledged that there exists a multiplicity of measures to prevent overweight or obesity such as encouraging physical activity [3], reducing alcohol intake [4] and reducing daytime napping [5]. However, the fundamental cause of overweight and obesity is an energy imbalance between calories consumed and calories expended.

At the individual level, people can

- Limit their energy intake from total fats and sugars [1];
- Increase their consumption of fruit, vegetables, legumes, whole grains and nuts [1]
- Engage in regular physical activity [1, 3].

Individual responsibility can only have its full effect where people have access to a healthy lifestyle. Therefore, at the societal level it is important to undertake policies that support individuals to engage in regular physical activity and make healthier dietary choices [1].

Consuming too much sugar and too many foods and drinks high in sugar content, causes not only tooth decay [6] but also weight gain [7] leading to subsequent overweight and obesity related morbidities. A recent report from the United Kingdom (UK) [8] proposed the following multifaceted approach in order to decrease calorific consumption from sugars.

- A gradual reduction of sugar content in everyday food and drink products, combined with reductions in portion size.
- Price increases of a minimum of 10–20% on high sugar products through the use of a tax or levy such as on full sugar soft drinks [9].
- Reduction in price promotions in all retail outlets including supermarkets, stores, restaurants and takeaways.
- Reduction in advertisements for high sugar food and drink products to children and adults.
- Implementation of public sector catering standards to ensure provision and sale of healthier food and drinks in hospitals, leisure centers etc.
- Encourage health promotion by providing practical steps to help individuals lower their own and their families’ sugar intake.

Just as in other developed and developing countries, Israelis are not immune from being overweight or obese and their related morbidities [10].

This paper is a response to the request of the Minister of Health, to provide an estimate as to the potential reduction in mortality, morbidity and health care costs if a national program (containing many elements similar to that proposed in the UK [8]), but with the addition of labelling of products with high sugar content [11], to reduce calorific intake from sugar consumption is adopted and implemented in Israel.

## Methods

### Prevalence

Estimates of the gender, religion (Jews and non-Jews) and age-specific (20–64, 65+) measured prevalence rates of overweight and obesity in Israel in 2016, were based on extrapolations and interpolations of data from a number of national surveys on self-reported [12–14] and measured [15, 16] rates of overweight and obesity.

### Relative risks

Cause and gender-specific data on relative risks (along with 95% Confidence Intervals) for 16 diagnoses were obtained from a Canadian cost of illness study based on meta-analyses [17] and from a more recent meta-analyses for type II diabetes [18], non-alcoholic fatty liver disease [19] and gout [20], the latter two diagnosis specific risks being based on average BMI levels of 27.3 and 33.6 in Israeli overweight and obese persons respectively [21].

### Population attributable fraction (PAF)

The gender and diagnosis specific PAF, were calculated, for both the prevalence of overweight and obesity in turn using the following standard formula:-

$$ \mathrm{P}\mathrm{A}\mathrm{F} = \frac{\mathrm{prevalence} \times \left(\mathrm{R}\mathrm{R}\ \hbox{--}\ 1\right)}{\left[\mathrm{prevalence} \times \left(\mathrm{R}\mathrm{R}-1\right) + 1\right]} $$

The resultant PAF were then aggregated across genders and combined for overweight and obesity in order to arrive at a composite diagnosis specific PAF.

### Hospital expenditures

Age, gender and cause specific data on days hospitalization in general hospitals for persons over 20 in 2016 were estimated by applying the average age, gender and cause specific hospitalization rates in persons aged 20+ from 2010 to 2013 (Personal communication, Tziona Haklaii and Nehama Goldberger of the Health Ministry’s Information Division) to the age and gender specific population data for 2016, which in turn was based on extrapolations of data from 2013 to 2015 [22–24]. Total general hospital costs for diseases associated with overweight and obesity were calculated by multiplying the disease specific utilisation data by the per day costs (2127–2613 NIS) associated with their respective departments [25] and the disease specific utilization of intensive care units costing around 5852 NIS per day [26]. The resultant figure was then multiplied by a factor of 1.33 [23] to take into account utilisation of geriatric hospitals, rehabilitation and convalescent facilities as well as associated research costs (which are an integral part of providing quality medical care in hospitals).

### Direct health expenditures

Cause specific costs of out of hospital pharmaceutical use and other services (eg; ambulatory, emergency room, out- patient visits etc.) were estimated by applying cost ratios from a Canadian study [17] to the Israeli based hospital expenditures

$$ \mathrm{Total}\ \mathrm{Direct}\ \mathrm{Costs} = \mathrm{Hospital}\ \mathrm{Costs} + \mathrm{Out}\ \mathrm{of}\ \mathrm{Hospital}\ \mathrm{Pharmaceutical}\ \mathrm{Costs} + \mathrm{Other}\ \mathrm{service}\ \mathrm{Costs} $$

### Productivity losses and other indirect costs

Estimates of indirect costs included mainly productivity losses, due to increased absenteeism and presenteeism (i.e. workers coming in to work, but with impaired productivity due to their health condition), in addition to informal carers costs and transport costs. Imputed human capital costs from premature mortality were excluded as these are in effect “virtual” costs, but frictional employment costs (i.e.; retraining substitute workers to replace the deceased worker) were included. Estimates of the ratio of indirect costs relative to health service costs were based on international published literature [17, 27–41], where information was available that enabled distinction between human capital valuations of mortality and other indirect costs. This ratio was then multiplied by the calculated direct health services costs in order to provide cause specific estimates for indirect costs resulting from overweight and obesity. Since categorizations of indirect costs are not always homogeneous, we used a sensitivity analysis based on the semi-interquartile range for confidence limits around our median estimate.

$$ \mathrm{Total}\ \mathrm{Societal}\ \mathrm{Costs} = \mathrm{Direct}\ \mathrm{Health}\ \mathrm{Expenditures} + \mathrm{Productivity}\ \mathrm{Losses} + \mathrm{Care}\ \mathrm{Costs} + \mathrm{Transport}\ \mathrm{Costs}. $$

The disease-specific product of these expenditures and PAF estimated the various cost of these diseases.

### Dental fillings

Israelis consume on average 22.9 kg per capita (62.7 g. per day) of sugar annually [23], this provides 12.45% of all calorific intake in Israel [23] similar to the figure of 12.06% in the UK [7]. A cross-section of international data [42], enabled the calculation of the prevalence of caries attributable to sugar, based on regression coefficient slopes of 0.02 (intercept of no sugar consumption estimates 2.13 caries) in milk teeth (in persons aged 6.5 years old) and 0.04 (intercept of 0.06) caries per gm. per person per day in adult teeth (in persons aged 12.5 years old), This figure was then divided by the amount of sugar consumed by the two groups in order to estimate the annual caries incidence per exposed tooth year attributable to sugar consumption and other causes in Israel. In keeping with guidelines from the UK, we only included the impact of sugar consumption and changes in the incidence of dental caries in children and young adults (under 18 years of age), because the development of dental caries depends on life-long exposure to risk factors [43].

The following formula was used for milk and adult teeth in turn, aggregated up to 18 years old:-

$$ \begin{array}{l}\mathrm{Incidence}\ \mathrm{o}\mathrm{f}\ \mathrm{caries}\kern0.5em  = \mathrm{Incidence}\ \mathrm{per}\ \mathrm{t}\mathrm{o}\mathrm{o}\mathrm{t}\mathrm{h}\ \mathrm{year} \times \mathrm{number}\ \mathrm{o}\mathrm{f}\ \mathrm{t}\mathrm{o}\mathrm{o}\mathrm{t}\mathrm{h}\ \mathrm{year}\mathrm{s}\ \mathrm{in}\ \mathrm{population} \times \\ {}\left(100\% - \%\ \mathrm{decrease}\ \mathrm{due}\ \mathrm{t}\mathrm{o}\ \mathrm{f}\mathrm{luoridation}\right)\end{array} $$

Assuming fluoride in the water supply reduces caries by 35.5% (95% CI 25.7–45.3%) in milk teeth and 26.4% (95% CI 16.4–36.6%) in adult teeth [44].

### Cost of fillings

Cost of fillings were based on sick fund specific costs (ranging from 162 to 193 NIS for a single surface and 253–596 NIS for multiple surfaces) in proportion to their membership [45] and private dental costs (150–600 NIS), assuming 40% were carried out by independent private dentists.

### Mortality

Gender and cause specific data on mortality for persons over twenty years old in 2016 were estimated by applying the average age, gender and cause specific mortality rates in persons aged 20+ from 2009 to 2013 (Personal communication, Tziona Haklaii and Nehama Goldberger of the Health Ministry’s Information Division) to the aforementioned age and gender specific population data for 2016 [22–24].

These cause and gender specific deaths were then multiplied by the cause and gender specific PAFs in order to estimate the number of deaths attributable to overweight and obesity in 2016.

### Potential Years of Life Lost (PYLL)

Extrapolations of age and gender specific life expectancies to 2016 [22, 23] were multiplied by age-gender and cause specific mortality data in order to calculate the cause specific PYLL attributable to overweight and obesity.

### Disability Adjusted Life Years (DALY)

Age- and gender-specific disability weights, used by the Ministry of Health, were applied to the life expectancies in 2016 [22, 23] in order to calculate the age and gender specific Healthy Adjusted Life Expectancy (HALE), subject to a 3% per annum discount rate. These HALEs were subsequently multiplied by the age-gender and cause specific mortality data from 2010 to 2013 (Personal communication, Tziona Haklaii and Nehama Goldberger of the Health Ministry’s Information Division) in order to calculate the cause specific DALY loss due to mortality.

### DALY loss due to morbidity

Due to lack of cause specific disability weights, we assumed that each days hospitalization resulted in a disability weight of 0.3 (varied using a sensitivity analysis between 0.2 and 0.4), which was then multiplied by the age-and gender specific hospitalization rates adjusted by the Ministry of Health’s age- and gender-specific disability weights of health people in order to estimate the DALY losses due to morbidity.

### Sugar reduction

Data from a recent UK modelling study [8] was used to estimate the effect on mortality, morbidity and costs of reducing calorific consumption of sugar from 12.45 to 10% in 5 years (a feasible short-term goal) or to 5% in fifteen years (a harder to attain long-term goal) by means of a multi-faceted National program.

### Cost utility analysis

The basic formulae used for calculating the cost per averted DALY loss is:-

$$ \mathrm{Cost}\ \mathrm{per}\ \mathrm{averted}\ \mathrm{DALY}\ \mathrm{loss} = \frac{\mathrm{Cost}\ \mathrm{of}\ \mathrm{in}\mathrm{tervention}\ \hbox{--}\ \mathrm{Tax}\ \mathrm{revenues} - \mathrm{Savings}\ \mathrm{in}\ \mathrm{costs}}{\mathrm{Averted}\ \mathrm{DALY}\ \mathrm{loss}\mathrm{es}} $$

where:-

*From the health services perspective:*

$$ \mathrm{Savings}\ \mathrm{in}\ \mathrm{costs} = \mathrm{Reduction}\ \mathrm{in}\ \mathrm{direct}\ \mathrm{treatment}\ \mathrm{costs} $$

*From the Societal perspective:*

$$ \mathrm{Savings}\ \mathrm{in}\ \mathrm{costs} = \mathrm{Reduction}\ \mathrm{in}\ \left(\mathrm{direct}\ \mathrm{treatment} + \mathrm{productivity}\ \mathrm{losses} + \mathrm{other}\ \mathrm{in}\mathrm{direct}\ \mathrm{costs}\right). $$

$$ \mathrm{Averted}\ \mathrm{DALY}\ \mathrm{losses} = \mathrm{Averted}\ \mathrm{DALY}\ \mathrm{losses}\ \mathrm{due}\ \mathrm{t}\mathrm{o}\ \mathrm{morbidity}\ \mathrm{and}\ \mathrm{mortality}\ \mathrm{decreases}. $$

All costs are presented in new Israeli shekels (NIS) at 2016 price levels. (at an exchange rate of 3.86 NIS to US dollar). Israel has no official policy guidelines for defining whether or not a health intervention is cost-effective. Affordability of interventions is a function of the amount of resources available in a country as proxied by its Gross Domestic Product (GDP) per capita. Therefore, WHO guidelines were used, which define an intervention as being very cost-effective [46] if the cost per DALY averted is less than the GDP per capita of 136,907 NIS ($35,427) in Israel [22–24, 47].

Estimated sugar tax revenues were based on recent UK estimates of 520,000,000 lb sterling per annum from just taxing soft drinks [48] adjusted for differences in population size and GNP per capita.

## Results

Table 1 shows the high estimated prevalence of overweight and obesity in 2016 for both genders aged 20–64 and the even higher prevalence in the over 65 s, especially amongst non-Jewish females. There are around 2,056,000 overweight and 1,719,000 obese adults in Israel.

**Table 1:** Overweight and obesity prevalence by gender, religion and age (2016)

	Overweight	Obese
20–64		
Jewish males	32.7%	21.9%
Jewish females	23.2%	19.5%
Non-Jewish males	32.5%	24.1%
Non-Jewish females	27.3%	29.6%
65+		
Jewish males	43.5%	31.2%
Jewish females	30.8%	27.8%
Non-Jewish males	43.2%	34.3%
Non-Jewish females	36.2%	42.2%

Disease specific relative risks for overweight and obesity are especially elevated for type II diabetes (both genders), pulmonary embolisms (both genders), osteoarthritis (males) and coronary arterial disease (females) (Table 2).

**Table 2:** Disease-specific relative risks for overweight and obese persons by gender

	Overweight		Obese		Ref.
	Male	Female	Male	Female	
Asthma	1.20	1.25	1.43	1.78	[14]
Breast cancer	1.00	1.08	1.00	1.13	[14]
Congestive heart failure	1.31	1.27	1.79	1.78	[14]
Colorectal cancer	1.51	1.45	1.95	1.66	[14]
Coronary artery disease	1.29	1.80	1.72	3.10	[14]
Diabetes type II	2.20	3.60	6.05	11.15	[14, 15]
Endometrial cancer	na	1.53	na	3.22	[14]
Gallbladder disease	1.09	1.44	1.43	2.32	[14]
Gout	2.02	1.79	3.70	2.95	[20]
Hypertension	1.28	1.65	1.84	2.42	[14]
Kidney cancer	1.40	1.82	1.82	2.64	[14]
Non-alcoholic fatty liver	1.30	1.52	4.09	4.78	[19]
Esophageal cancer	1.13	1.15	1.21	1.20	[14]
Osteoarthritis	2.76	1.80	4.20	1.96	[14]
Ovarian cancer	na	1.18	na	1.28	[14]
Pancreatic cancer	1.28	1.24	2.29	1.60	[14]
Prostate cancer	1.14	1.00	1.05	1.00	[14]
Pulmonary embolism	1.91	1.91	3.51	3.51	[14]
Stroke	1.23	1.15	1.51	1.49	[14]

Estimated direct costs of comorbidities related to overweight and obesity in Israelis aged over 20 years old were around 4763 million NIS of which hospital care accounted for 44.8% and pharmaceutical consumption outside of hospitalization 24.2% (Appendix 1).

Since the PAF for Type II Diabetes exceeded 100%, we constrained its PAF to be an arbitrary 90%. Around 6402 people (95% CI 3296–8760) died in 2016 (Table 3) as a result of conditions associated with them being overweight (2315 deaths: 95% CI 695–3535) or obese (4086 deaths: 95% CI: 2600–5225). The leading causes of death were Coronary Artery Disease which accounted for 2207 (95% CI: 1763–2649) fatalities (34.5% of all attributable deaths), Type II Diabetes (13.2%), Congestive Heart Failure (12.8%), Stroke (10.5%) and Colorectal Cancer (9.5%).

**Table 3:** Deaths attributable to overweight or obesity in adults over 20 years old by diagnosis (2016)

	Deaths	Attributable to obesity or overweight	(%)	Attributable to overweight	Attributable to obesity
Coronary artery disease	4949	2207	34.5%	796	1410
Diabetes type II	941	847	13.2%	315	532
Congestive heart failure	2279	822	12.8%	276	546
Stroke	2742	671	10.5%	224	446
Colorectal cancer	1456	609	9.5%	262	347
Hypertension	846	399	6.2%	142	257
Pancreatic cancer	938	344	5.4%	99	245
Pulmonary embolism	144	111	1.7%	41	70
Kidney cancer	232	110	1.7%	44	66
Breast cancer	1111	78	1.2%	31	48
Endometrial cancer	92	55	0.9%	15	40
Ovarian cancer	292	43	0.7%	18	26
Prostate cancer	394	35	0.5%	27	8
Asthma	116	32	0.5%	10	22
Esophageal cancer	143	18	0.3%	8	10
Gallbladder disease	30	10	0.2%	3	7
Non-alcoholic fatty liver	16	6	0.1%	2	4
Gout	4	2	0.03%	1	1
Osteoarthritis	3	2	0.03%	1	1
Total	16,729	6402	100%	2315	4086

Each fatality lost an average of 12.4 years (PYLL) or 7.1 (discounted) years of HALE, with an average associated disability weight (DW) of 0.43, which reflects the high percentage of deaths in the 75–84 (25%) and 85+ age groups (41%) when functional deterioration has set in even amongst healthy persons.

Around 268,206 (95% CI: 135,362–354,252) hospital days were attributable to Overweight (98,545) and Obesity (169,464), accounting for 16.1% (95% CI: 4.5–23.7%) and 27.6% (95% CI: 17.5–34.0%) respectively of the 612,844 hospital days associated with related diagnoses (Appendix 2).

Coronary Artery Disease (21.5%) Type II diabetes (21.1%), and Hypertension (20.4%) account for over three-fifths of the 2185 (95% CI: 878–2667) million NIS direct costs (Table 4) related to overweight (802 million NIS : 95% CI 51–1122 million NIS) and obesity (1383 million NIS : 95% CI 827–1545 million NIS).

**Table 4:** PAF and direct costs (NIS) attributable to overweight and obesity by diagnoses - Israeli adults (2016)

	PAF overweight	PAF obesity	Total cost	Attributable to overweight & obesity	
Coronary artery disease	16.1%	28.5%	1,055,915,767	470,784,150	21.5%
Diabetes type II	33.5%	56.5%	511,313,763	460,182,387	21.1%
Hypertension	16.8%	30.4%	943,186,656	445,157,136	20.4%
Congestive heart failure	12.1%	24.0%	462,954,791	166,984,682	7.6%
Osteoarthritis	31.1%	35.8%	247,895,278	165,852,084	7.6%
Stroke	8.2%	16.3%	490,337,015	119,962,933	5.5%
Colorectal cancer	18.0%	23.8%	220,788,825	92,322,002	4.2%
Pulmonary embolism	28.2%	48.6%	93,633,779	71,915,718	3.3%
Asthma	8.4%	19.1%	260,546,602	71,698,574	3.3%
Gallbladder disease	9.4%	23.7%	115,622,301	38,181,949	1.7%
Kidney cancer	19.1%	28.5%	46,068,752	21,944,698	1.0%
Pancreatic cancer	10.6%	26.1%	52,536,885	19,257,766	0.9%
Endometrial cancer	16.3%	44.2%	28,576,252	17,298,101	0.8%
Breast cancer	2.8%	4.3%	124,719,472	8,786,692	0.4%
Prostate cancer	6.9%	2.0%	49,259,925	4,358,259	0.2%
Ovarian cancer	6.0%	8.8%	28,750,951	4,263,974	0.2%
Esophageal cancer	5.7%	7.0%	23,724,293	3,015,682	0.1%
Non-alcoholic fatty liver	14.6%	22.4%	4,413,971	1,633,711	0.1%
Gout	31.4%	16.1%	3,409,277	1,618,618	0.1%
Total			6,512,219,714	2,185,219,115	100.0%

The ratio of indirect to health service costs from the literature had a median value of 72.2% (semi-interquartile range 44.7–130.0%), giving an estimate of 1578 (95% CI: 977–2841) million NIS for the indirect costs. Therefore, the total costs to society attributable to overweight (1381: 95% CI 74–2581 million NIS) and obesity (2382: 95% CI 1197–3554 million NIS), amount to around 3763 (95% CI: 3163–5027) million NIS annually.

The estimate of caries prevalence per person attributable to sugar consumption was 1.25 (in children aged 6.5 years) and 2.51 (in children aged 12.5 years). This translates into an annual per capita caries incidence attributable to sugar of 0.21 for milk teeth and 0.39 for adult teeth respectively (Appendix 3). There is an estimated need for 1.67 million fillings annually in persons under 18 years old, costing around 374 million NIS (at an average cost of 224 NIS per filling). Sugar consumption was estimated to account for around 70.6% of all caries (and hence potential fillings) in this age group, at a cost of around 264 million NIS annually in Israel.

Hence, the direct and societal costs attributable to obesity, overweight and sugar consumption are 2449 million and 4027 million NIS respectively (both costs including 264 million caries costs attributable to sugar consumption), representing 0.21 and 0.35% of GDP respectively.

## Discussion

In contrast to deaths which are clearly attributable to a given causality (such as motor vehicle collisions, suicides, falls, fires, drowning etc..), deaths due to pollution and to personal behaviours, such as smoking, sedentariness and nutritional habits are harder to identify. Despite this difficulty, obesity and overweight have been implicated as risk factors for many causes of death [17, 49, 50].

The estimate of 6402 deaths annually attributable to overweight and obesity (around 15% of all deaths) is over 17 times the magnitude of suicides, 18 times that of road accident fatalities and around 50 times that of homicides in Israel [23]. The direct cost of treating morbidity from overweight and obesity, is around 2.18 billion NIS, equivalent to 0.19% (95% CI: 0.08–0.23%) of Israel’s GDP [23, 24] or 2.5% (95% CI: 1.0–3.9%) of all health expenditures. This represents an average annual cost of 391 NIS and 802 NIS respectively, for each obese or overweight Israeli aged 20 and over.

The total costs to society of treating morbidity from obesity and overweight, is around 3.8 billion NIS, equivalent to 0.32% (95% CI: 0.27–0.43%) of GDP. [17, 18], an average annual expenditure of 674 NIS and 1381 NIS respectively for each obese or overweight Israeli aged 20 and over. Due to the high burden of disease, introduction of programs to reduce sugar consumption are desirable.

### Impact of reducing sugar consumption

An as yet undefined, national program of reducing energy from sugar consumption from its current level of 12.45 to 10% will reduce damage from sugar related obesity (and overweight) by 3.7% over a five year time horizon [7] and reduce caries by 16.1%. This will annually, on average, prevent 237 (95% CI: 112–325) deaths, 9917 days hospitalization and around 180,000 fillings (Table 5). This will result in annual savings of direct treatment costs of around 121 million NIS (including 40 million NIS from caries reduction) and total societal costs of 180 million (95% CI: 118–294 million) NIS or 0.02% (95% CI: 0.01–0.03%) of GDP (Table 5).

**Table 5:** Average annual mortality, morbidity decreases and cost savings (NIS) by sugar consumption goals

		Lower 95% limit	Upper 95% limit
10% of energy from sugar in 5 years			
Averted:-			
Deaths	237	122	325
General hospital days	9917	4993	13,068
Caries	179,625	153,843	205,050
Savings (NIS):-			
Fewer fillings	40,276,302	34,495,259	45,977,166
Overweight & obesity treatment	80,854,527	47,172,679	143,248,124
Total direct costs (NIS):	121,130,829	81,667,937	189,225,290
Indirect costs	58,375,715	36,166,004	105,129,999
Total societal costs (NIS)	179,506,544	117,833,941	294,355,290
% of per capita GDP	0.02%	0.01%	0.03%
5% of energy from sugar in 15 years			
Averted:-			
Deaths	494	254	677
General hospital days	20,699	10,423	27,277
Caries	336,484	288,187	384,111
Savings (NIS):-			
Fewer fillings	75,447,779	64,618,411	86,126,949
Overweight & obesity treatment	168,767,366	98,463,364	299,001,300
Total direct costs (NIS):	244,215,145	163,081,775	385,128,250
Indirect costs	121,847,423	75,489,171	219,437,473
Total Societal Costs (NIS)	366,062,568	238,570,946	604,565,723
% of per capita GDP	0.03%	0.02%	0.05%

If a more ambitious target of reducing consumption to 5% is achieved over a 15 year period, sugar related obesity (and overweight) will decrease by 7.7% [7] and caries by 49.1%. This means that annually an average of 494 (95% CI: 254–677) deaths, 20,699 days hospitalization and 336,000 fillings will be prevented, resulting in savings of around 244 million NIS in direct costs (including 75 million NIS in caries reduction) and societal costs of 366 million (95% CI: 239–605 million) or 0.03% (95% CI: 0.02–0.05%) of GDP.

### Cost utility analysis of reducing sugar consumption

Assuming the imposition of an intervention program will succeed in reducing energy consumption from sugar to 10% over 5 years, there will be a saving of 1184 lives and 14,703 PYLL, giving a total of 8425 averted discounted DALYS. Since 99.7% of these DALYS are due to mortality gains, varying the disability weight of hospitalization between 0.2 and 0.4 will have an insignificant effect on the results.

Imposing a tax on sugar products could be part of the multifaceted approach to reducing sugar consumption. Taxing soft drinks could annually generate around 280 million NIS of income (based on adjusted UK estimates). In the UK such revenues are earmarked to improve primary school sports facilities. In Israel, around 24 million NIS of the revenues annually could be earmarked to add 100 full time equivalent dietician posts (including office facilities) to help implement other parts of the multifaceted program to reduce sugar consumption. Over 5 years this hypothetical intervention will for an intervention cost of around 120 million NIS, generate 1400 million in tax revenues, save 606 million NIS in direct treatment costs and a further 292 million in indirect costs, resulting in a net (cost-) saving of 2178 million NIS.

However, the Health Minister is reluctant to impose a sugar tax, on the grounds that such a tax will raise consumer prices in a regressive fashion [11]. In this scenario, the intervention will still be cost saving to the tune of 778 million NIS. While any future imposition of a sugar tax will impose only a small legislative cost, lessons should be learned from the recent experience in New York of the practical problems of imposing such a tax, especially in that it should be based on a per calorie as opposed to per volume formula [50].

These estimates, of damage from sugar consumption, should be regarded as preliminary as they can be improved upon if the following data ever becomes available:

i) Israeli-based cause and age-specific relative risks, thus allowing calculation of PAF due to obesity and overweight in Israel.
ii) Israeli based disease specific costs and utilization rates of geriatric hospitals, out of hospital pharmaceutical and other care costs (home helps, physiotherapy, ambulatory doctor visits etc.).
iii) Israeli-based cause specific estimates of work productivity losses and other indirect costs.
iv) Estimates of disability weights relating to morbidity in an out-of hospital setting. This would correct for the underestimation of DALYS averted (and subsequent overestimation of Costs per DALY averted) since only estimates for morbidity losses during the period of hospitalisation were included in the model.

Gains from reduced caries might also be overestimated to the extent that some people in Israel are already exposed to fluoride via toothpaste and their natural water supplies. On the other hand, estimates of the costs of treating caries and hence potential savings attributable to reductions in sugar consumption can be considered as being very conservative as they do not include any costs incurred by persons aged 18 years and over, who will almost certainly benefit from a reduction in sugar consumption [51].

Further underestimations of averted DALYS and averted treatment costs arose because the model was based solely on the relative risks of overweight and obese persons and was unable (because of lack of data) to take into account the impact of reductions in sugar consumption in persons of normal weight. For example, many new onset cases of type II diabetes occur in persons of normal anthropometric proportions who would also benefit from reduction in sugar consumption [52].

All the estimates are subject to the important caveat that any achieved calorific reduction due to decreased sugar consumption is not compensated for by increased calorific intake of other (non-sugar) foods. But even in the event that a compensating rise in calorific consumption occurs (resulting possibly in an isocaloric situation), some health benefits are likely to still be generated as “not all calories are equal” since there is evidence that the quality of fat and carbohydrate can play a more important role than the quantity [53]. The resultant diet that is lower in sugar (carbohydrates) is likely be healthier than the initial high in sugar diet, especially with respect to type II diabetes risk factors in young people [54]. If full calorific compensation occurs, then the mortality and morbidity gains estimated in this paper will be around 74% lower [55], resulting in there being a gain of 2190 DALYs and a net saving of only 113 million NIS in the scenario where no tax is imposed on sugar.

Reducing energy from sugar consumption from its current level of 12.45 to 10%, over a five year time horizon is considered to be a very reasonable and attainable short-term goal. Over these 5 years, this would save 2178 million NIS in costs (778 million NIS if no taxes are imposed) as well as 1184 lives. Achieving a reduction to 5% over a 15 year period would be a far harder goal to achieve, but the rewards in terms of decreased mortality, morbidity and expenditures would be greater.

It is highly likely that any package of interventions, with or without the imposition of taxes on sugar, will be cost-saving (i.e. supplying quality adjusted life years at no additional net cost), since costs savings from morbidity reductions (and possible tax revenues) will exceed the costs of the intervention. This will still be true if we view the results just from just the direct costs of health services perspective. Finally, it should be noted that other many other interventions available to reduce overweight and obesity are available, that have also been shown to be cost-saving or very cost effective [21, 56].

## Conclusions

Sugar consumption causes a huge monetary and mortality burden. Estimates of potential decreases in this burden justify the current prioritisation given by the health minister of creating and implementing a national program to reduce sugar consumption, which is likely to be cost-saving (ie: averted treatment costs will exceed intervention costs).

## Appendix 1

**Table 6:** Direct costs (NIS by type) of comorbidities related to overweight and obesity in Israeli adults (2016)

	Hospital care	Other care	Drugs excl hosp	Total cost	
Coronary artery disease	591,658,411	242,594,993	221,662,363	1,055,915,767	22.2%
Hypertension	61,099,650	395,143,788	486,943,218	943,186,656	19.8%
Diabetes type II	206,869,057	181,625,245	122,819,461	511,313,763	10.7%
Stroke	370,683,266	112,966,069	6,687,680	490,337,015	10.3%
Congestive heart failure	296,083,592	105,115,078	61,756,121	462,954,791	9.7%
Asthma	41,796,206	109,329,885	109,420,510	260,546,602	5.5%
Osteoarthritis	108,937,052	75,063,697	63,894,529	247,895,278	5.2%
Colorectal cancer	144,207,495	64,220,043	12,361,287	220,788,825	4.6%
Breast cancer	47,387,005	56,090,016	21,242,451	124,719,472	2.6%
Gallbladder disease	77,257,439	30,853,722	7,511,140	115,622,301	2.4%
Pulmonary embolism	52,141,557	23,642,864	17,849,358	93,633,779	2.0%
Pancreatic cancer	35,098,638	14,772,528	2,665,719	52,536,885	1.1%
Prostate cancer	22,111,658	21,229,074	5,919,193	49,259,925	1.0%
Kidney cancer	26,858,616	15,474,832	3,735,304	46,068,752	1.0%
Ovarian cancer	19,039,091	8,197,386	1,514,473	28,750,951	0.6%
Endometrial cancer	14,533,230	10,957,594	3,085,428	28,576,252	0.6%
Oesophageal cancer	16,018,731	6,573,203	1,132,359	23,724,293	0.5%
Non-alcoholic fatty liver	2,054,218	1,341,950	1,017,803	4,413,971	0.1%
Gout	1,586,644	1,036,499	786,134	3,409,277	0.1%
Total	2,135,421,555	1,476,228,468	1,152,004,532	4,763,654,555	100.0%

## Appendix 2

**Table 7:** Hospital days attributable to overweight and obesity in Israeli adults (2016)

	Days	Overweight	Obesity	Normal or underweight
Coronary artery disease	169,800	27,316	48,390	94,094
Diabetes type II	59,369	19,962	33,470	5937
Congestive heart failure	84,973	10,285	20,364	54,324
Stroke	106,382	8707	17,320	80,355
Osteoarthritis	31,264	9733	11,183	10,347
Colorectal cancer	41,386	7451	9855	24,081
Pulmonary embolism	14,964	4215	7278	3471
Hypertension	17,535	2942	5334	9259
Gallbladder disease	22,172	2075	5247	14,850
Pancreatic cancer	10,073	1063	2629	6381
Kidney cancer	7708	1474	2198	4036
Asthma	11,995	1008	2293	8694
Endometrial cancer	4171	680	1844	1646
Breast cancer	13,600	375	583	12,641
Ovarian cancer	5464	329	482	4654
Esophogal cancer	4597	263	321	4013
Prostate cancer	6346	436	125	5784
Non-alcoholic fatty liver	590	86	327	177
Gout	455	143	221	91
Total	612,844	98,545	169,464	344,835

## Appendix 3

**Table 8:** Relationship between caries incidence attributable to sugar consumption in Israel

	Milk teeth	Adult teeth
Age of child (years)^a^	6.5	12.5
Caries with zero sugar (intercept)^a^	2.13	0.06
Caries per gm per person day^a^	0.02	0.04
Caries prevalence from sugar^b^	1.25	2.51
% of caries from sugar	37%	98%
Tooth years of exposure	94	72
Sugar consumption (Kgm)^c^	137	149
Caries per Kgm sugar consumption	0.009	0.017
Annual caries incidence per person^d^	0.21	0.39

Notes:-

^a^based on regressions from international cross-sectional study [36]

^b^based on Israeli consumption of 62.7 g/day

^c^from 6 months to 6.5 years for milk teeth and 6.0 years to 12.5 years for adult teeth

^d^attributable to sugar

## Abbreviations

BMI: Body Mass Index
DALY: Disability adjusted life year
GDP: Gross Domestic Product
HALE: Healthy Adjusted Life Expectancy
NIS: New Israeli shekel
PAF: Population attributable fraction
PYLL: Potential Years of Life Lost
RR: Relative risk
UK: United Kingdom of Great Britain and Northern Ireland
WHO: World Health Organization
